# The Publicness of Pandemic Security and the Shortcomings of Governmentality

**DOI:** 10.1007/s41125-022-00084-w

**Published:** 2022-10-11

**Authors:** Andreas Langenohl

**Affiliations:** 1 Department of Sociology and Collaborative Research Centre/Transregio 138 “Dynamics of Security”, Justus Liebig University Giessen, Giessen, Germany; 2 School of Government Studies, North-West University, Potchefstroom, South Africa

**Keywords:** Biopolitics, COVID-19 pandemic, Foucault, Michel, Governmentality, Public sphere, Securitization theory, Sovereignty

## Abstract

Employing the example of Germany within a European context, this paper argues that government responses to the pandemic relied too much on the biopolitical governance of populations, and too little on the symbolic governance of public spheres. Based on an analysis of policy documents and their medial representation, it is found that the politics of pandemic security is focused on the regulation of population aggregates and movements (social distancing, lockdowns, border closings, etc.), resembling a quasi-Foucaultian notion of biopolitical governmentality. Confident that the crisis can be handled through a classical apparatus of security through self-conduct within an imaginary of stochastic aggregation of the social, these modes of governance paid virtually no attention to non-stochastic social aggregates, such as those which can be observed in public spheres. Yet these aggregates produced massive mobilizations against the politics of pandemic governance in liberal democracies, in the streets and on the internet. In conceptual terms, these mobilizations can be understood as an insistence on sovereign power, in Foucault’s sense, yet ‘from below’: They reinvigorate the dramatic public, as opposed to the inconspicuous circulation, as the site for claiming attention, legitimacy, and potentially disruption—in other words, for claiming sovereign power. In the final analysis, a major security problematic can be seen in the failure of the politics of governmentality to be insensitive to the politics of sovereignty.

## Introduction

Issues of public security during the COVID-19 pandemic cover a much more encompassing terrain than merely public health.^1^ They relate, crucially, to maintaining public order, keeping the political economy going, and to the safeguarding of essential social functions, addressed, for instance, as critical infrastructures. Moreover, especially in European contexts, protests against pandemic containment measures attracted concerns about public security. The record from Germany lists politicians being bullied by protesters,^2^ demonstrators using children as shields against police interventions,^3^ and an individual being murdered because he insisted that a gas station customer wear a mask, with the suspected perpetrator finding his act justified in digital messenger networks.^4^ Diverse as these numerous and non-overlapping events are, they all can be considered public challenges to the German government’s anti-pandemic policies.

This publicness of pandemic security and insecurity is an important vantage point from which to gauge the effects of governments’ Corona-related measures. In many contributions on how governments tried to steer through the pandemic, the notion of the ‘public’ is used in the sense of public opinion, that is, a set of attitudes among the population affirming or challenging the legitimacy of governments or states with respect to their responses to the pandemic (see, for instance, Chen and Fan 2022). However, the legitimacy and normative shape of ‘the public’ itself was one of the core aspects at stake in the pandemic, as lockdown measures were met with numerous protests that precisely claimed for themselves those public spaces that the anti-pandemic policies had evacuated. Indeed, the very meaning of ‘public life’ underwent changes through the pandemic (see in the example of Germany, the contributions in Hahn and Langenohl 2022). It is the argument of this paper that the protests directed against the anti-pandemic policies, and the violence that accompanied them, were interrelated with governments’ own role as agents of pandemic publicness.

I will focus on public manifestations of invocations of (in-)security that will help understand the cleavage and interrelation between government policies and anti-system protests. While such protests, especially in Germany, are not anything new, the pandemic brought highly diverse protesters to the streets who challenged, as openly and loudly as ever before, the legitimacy of government, state institutions, and important aspects of the lawful state. The publicness of pandemic security invites a consideration from the point of view of securitization theory. Because securitization theory analytically distinguishes between givens of (in-)security and social and political claims of (in-)security—often termed ‘securitization’—it is especially suited for analyzing complex security conditions that involve public statements, proclamations, and actions.

While mostly not put to this particular use, securitization approaches are instrumental in reconstructing the *effectiveness* of political securitization decisions and strategies. True, mostly the ‘referent objects’ of securitization are supposed to be more or less stable—namely, state institutions and national sovereignty, which constitutes a continuum with the ‘realist’ branch of International Relations (Wæver 1995). Yet, more recent research has shown that there is a plethora of potential referent objects of securitization, including the economy, the environment, cultural identity, and others (Buzan et al. 1998; Stritzel 2012; Diez et al. 2016). The pandemic confronted political actors and institutions with the necessity to make security prioritizations. These may be analytically reconstructed in terms of securitization decisions and strategies.

These decisions and strategies concern not only the type of object that is securitized, but also the modalities through which this is achieved. Securitization research has introduced and elaborated at length on different theoretical perspectives on the social and political constitution of security issues and concerns (see, for overviews, Wæver 2015; Balzacq et al. 2016; Langenohl 2019). These perspectives can be broadly aligned with the following theoretical branches: speech act theoretical approaches which conceive of securitization as a public speech act by an authorized figure that declares an emergency situation (Wæver 1995; Buzan et al. 1998); practice theoretical inroads, which investigate the activities and framing power of experts and professionals on security issues (Bigo 2006; Amoore 2013); and approaches that refer to categories developed by Michel Foucault in the context of his theorization of security (Foucault 2009) as a modality of ‘biopolitical’ or ‘governmental’ power, which focus on the ways that population dynamics are involved in the constitution of security issues (Collier 2009; Langley 2015). Relating to the pandemic, this internal differentiation of research on securitization enables one to categorize different invocations and constructions of existential threats to security along the lines of public discursive statements by politicians; the ostensibly less conspicuous work of experts and professionals engineering security apparatuses and drawing social boundaries; and a sort of politics relying on the dynamics of viral and human populations, that is, on a particular self-conduct of society together with the self-conduct of the (viral) problem.

Employing the example of Germany with a perspective on broader developments in Europe, this paper will argue that the government by and large chose strategies of governmentality, accompanied by some measures of disciplining, to the securitization of the pandemic. Beyond a mere application of Foucaultian categories to the current pandemic situation, the approach herein allows an analysis of the successes and failures of the different modalities of securitization by political actors and institutions. For instance, a security politics working mainly through governmentality might have different results, some of which may be aligned with the ‘intended’ results, while others would have to be classified as unintended or as wholly unforeseen. Hence, the use of Foucault’s categories may inform a policy evaluation.

This policy evaluation will reveal that securitization through governmentality failed in one crucial regard. Governments in Europe, and exemplarily the German one, through over-emphasizing a governmental approach to invoking and guaranteeing security during the pandemic, neglected other registers of securitization, especially the public drama of the “grammar of security” (Buzan et al. 1998, p. 33). Instead, this public register of securitization was appropriated by self-declaredly ‘anti-establishment,’ conspiracy theoretical, and right extremist social forces. Where public securitizations took place in the name of the political institutions, they were mainly conducted not by politicians but by health experts (instead of having them do their work, like security professionals often do, outside of the spotlight of public political communication, see Amoore 2013). As a consequence, official politics risked its public positionality as a credible agent of securitization.

The paper proceeds as follows: First, it introduces securitization theory as a way to conceive of the politics of security under pandemic conditions as a set of options for governments and political institutions to constitute and format security issues and agendas in different ways. The question is not just which policy field—like public health, political economy, education, critical infrastructures, etc.—is being securitized, but also through what modality of securitization this is taking place. Based on this theoretical discussion, and on the empirical basis of political documents and their medial representation, pandemic policies in Germany will be discussed as a case in which political institutions decided to securitize the pandemic through policies of governmentality and biopolitics—that is, policies that address the human population and its interactions with the viral population as the major vector in defining a security-related situation. The following section argues that, as an unintended consequence of this preoccupation with securitization along the lines of governmentality and biopolitics, another important strategy of securitization was neglected by political institutions that was then appropriated by the protest movement against the anti-Corona measures: namely, the policy of the “securitizing move” (Buzan et al. 1998, p. 25), the dramatic public speech act that serves to declare a state of emergency and to equip the utterer with authority and sovereignty. As a consequence, the securitization strategy of governmentality reached the limits of its political effectiveness, and political institutions confronted a situation in which they had given away the prerogative to talk sovereignty with forces politically diametrically opposed to them. A brief concluding section addresses the difficulty, yet maybe inescapability, to reconceptualize ‘sovereignty’ in times of the pandemic other than in terms inherited from the ‘realist’ register.

## Securitization: A Matter of Political Strategy

The pandemic confronted societies and politics worldwide with not just one, but multiple crises. Some of these crises were, depending on the governments, institutional contexts, pandemic phases, and framing devices, represented as large-scale existential crises. Some governments and politicians declared or demanded national states of health emergency or labeled other countries zones of high risk depending on a set of medical quantifiers. Others warned against the looming economic consequences of public life being shut down, or of the coming ‘educational catastrophe’ as a result of school closings. Still others were, at least initially, reluctant to frame the entire issue in terms of security at all (e.g., UK’s Boris Johnson, then U.S. president Donald Trump, Hungary’s Viktor Orbán, and Jair Bolsonaro in Brazil). The very pluralism of the responses to the pandemic cannot be explained by the local specificities of the epidemic dynamics alone, though. Instead, its causes have to be sought in the malleability of the very category of ‘security,’ and in the multitude of ways that threats to security can be politically and socially constructed. If the pandemic has shown one thing with respect to security, it is that the spread of a virus may be connected (or not) to a very heterogeneous set of existential issues that surpass the realm of public health.

Constructivist research into security issues is well prepared for an analysis of this kind of scenario. Since the 1980s, it has repeatedly suggested that matters of security are not pre-given through the very substrate of the security issue itself, but instead have to be politically and socially constituted as existential threats that demand a response. In line with this approach to studying security, this paper argues that any analysis of the state of pandemic-related security and of the effects of related political measures must start out with an analysis of the ways that the pandemic, and aspects connected with it, were politically and socially constructed as threats—that is, to use the technical term, how they were ‘securitized.’

At the same time, this kind of analysis faces the complicated issue as to how exactly to conceive of such processes of social and political constitution of security issues and agendas. The way that the debate in constructivist security research, especially in ‘securitization theory,’ unfolded indicates that, over time, conceptual layers have been added to the original conception of securitization as a speech act that maneuvers a given political issue or question from the realm of ‘ordinary’ politics into the realm of exceptionalism (Wæver 2004; Balzacq et al. 2016; Langenohl 2019). For instance, the speech act theoretical model was criticized for not paying enough attention to the concrete circumstances of securitization (Balzacq 2005), for not accounting for the plurality of discursive constructions that may constitute security issues (Stritzel 2012; Salter 2008), or for neglecting the dimension of security-related practices operating beneath the radar of explicit public statements (Amoore 2013). Still other approaches have sought to analyze the constitution of security issues and agendas along the lines of Foucaultian categories, especially that of biopolitics and governmentality (Collier 2009; Collier and Lakoff 2008, 2015; Langley 2013, 2015; Opitz 2016).

It is in particular these latter approaches which almost automatically seem to lend themselves to an analysis of pandemic-related securitization, given their interest in the ways that security agendas are interwoven with political conceptions of populations and their statistical dynamics (be it in the realm of health, as in the work of Stephen Collier, or in that of finance, as in Paul Langley’s research). Yet, it would be too straightforward to deduce the framework with which to analyze the constitution of security issues from the essence of those issues themselves; that is, as in the given case, to justify a biopolitical analytical framework with the substrate of the pandemic as affecting the biological state of individuals and populations. Instead, the first lesson of securitization research—namely, that the process of securitization follows logics that need to be analytically separated from any attributions one may make regarding the ‘nature’ of the security issue itself—also holds with respect to the question of which analytical framework one chooses for such an analysis.

As has been mentioned, the political responses to the pandemic demonstrated the variety of ways in which it could be securitized. This holds for the concrete issues and policy fields that were framed in terms of security, like public health, the political economy, or, recently, critical infrastructures which, as the newly formed expert council on the pandemic fears, might become understaffed as a consequence of a rapid increase in SARS-CoV-2 cases due to the Omicron variant.^5^ Yet it also holds for the ways and modalities that security issues are constructed, as analyzed within the different strands of securitization theory—for instance, through public political declarations, through expert and professional work, or through measures aiming to control the spread of the disease. In other words, while constructivist research into security has debated the different conceptualizations of securitization, it has also inadvertently arranged and prepared a set of deliberately political strategies to undertake securitization that is open for policymakers to choose from.

This adjacency of analytical categories and political options and strategies is nothing new in security-related research. For instance, Strategic Studies was the deliberate attempt to scienticize international relations (Amadae 2015). However, it is to be assumed that large swaths of constructivist security research, even as it turns against ‘realist’ or ‘essentialist’ understandings of security and insists on the contingency and self-logic of the process of securitization, have not closed themselves off from political address and functionality. On the contrary, their very institutionalization as peace research indicates a sought-after proximity to political decisions and rationalities. Hence, one can expect that governments, political actors, and institutions are reflective about processes of securitization and their potential consequences as described in the literature (see next section). This also means that securitization can be analyzed not only as a process of the constitution of security issues that needs to be analytically separated from those issues ‘themselves’, but also as an array of potential political *strategies* from which political actors and institutions—as may be assumed, more or less rationally and under given restrictions—choose. In other words, the politics of security may be strategic precisely *because* securitization cannot be reduced to the ‘nature’ of the security issue itself.

Seen in this light, the political responses to the pandemic become visible, analyzable, and evaluable as choices between different policies and modalities of securitization. This task will be embarked upon in the sections to follow, with the example of Germany within a wider European framework and based on official documents and news coverage.

## Pandemic Security Problematizations in Europe: Securing a Liberal Form of Life Through Infrastructures

On 18 March 2020, chancellor Angela Merkel took the unusual step to officially inform the public via television about the outbreak of the pandemic. In her speech, she described the current state of affairs as follows:

“Since German reunification, no, since the Second World War there was no challenge to our country in which all depends on our joint solidary agency. […] The point is to slow down the virus on its way through Germany. And for that it is existential to rely on the following: to shut down public life as far as possible. […] It depends on everybody. We are not condemned to passively accept the spread of the virus. We have a remedy: we have to considerably keep distance from one another. The virologists‘ counsel is unambiguous: no handshakes anymore, washing hands often and thoroughly, at least one and a half meters distance from the next person, and better hardly any contacts to the very old because they are particularly vulnerable.“^6^

In a similar manner, on the occasion of the Easter celebrations, on 11 April 2020 federal president Franz-Walter Steinmeier vied for citizens’ compliance with the strict rules of social distancing, appealing to the sense of solidarity among family members, relatives, friends, and persons in general:

“The major effort we are performing these days – we don’t perform it because an iron hand forces us to. But because we are an agile democracy of citizens who are aware of their responsibility [*mit verantwortungsbewussten Bürgern*]! A democracy in which we mutually extend our confidence [*einander zutrauen*] to listen to facts and arguments, to demonstrate reason, to do the right thing. A democracy in which every life counts and in which all depends on every single person: from the nurse to the federal chancellor, from the expert council of science to society’s visible and invisible pillars—at the supermarket checkouts, behind the wheels of buses and trucks, at the bakery [Backstube], on the farm or at the waste collection.“^7^

Rather than rhetorically invoking a state of exception, typical of a ‘securitizing move’ in the sense of the Copenhagen school (Williams 2003), both head of government and head of state addressed the population of Germany as a moral audience that can be convinced, won over, and made to accept extraordinary security measures. Their message was not so much that a vital threat was imminent, but rather that people in Germany ought to conduct themselves, due to their reasonableness and moral responsibilities, in particular ways. In short, it was an appeal to governmentality serving biopolitical rationales.

Correspondingly, the German government’s efforts to steer society through the multifaceted security crisis that the pandemic brought about can be described as a politics of infrastructural security (Langenohl and Westermeier 2022) which rested on governmental, as opposed to rhetorically securitizing, measures. They were concerned with calibrating the relationship between circulation and its interruption (as in the negotiations and oscillations regarding the governance of mobilities) and with the invocation of hubs of safety where large segments of the population were called upon to seek shelter from the pandemic and to shelter others (in particular, the ‘home’). These policies took the form of a balancing act between measures to interrupt the circulation of the virus and measures to safeguard those forms of circulation that were regarded as vital for society.

This regarded, first, the circulation of economic items such as commodities but also capital. The policies of lockdown, social distancing, border barriers, and travel bans were combined with the safeguarding of smooth traffic (especially road and rail) and, crucially, with the making available of financial funds for struggling businesses (Langenohl and Westermeier 2022). Second, the public health infrastructure was repeatedly addressed as being in continuous need of protection from overload. Exemplarily, Merkel explained in her government declaration on 26 November 2020 that such protection is required even under conditions of decreasing contact intensities^8^:

“Ladies and gentlemen, so far, the worst could be prevented—the overburdening of our public health system including the medical and, consequently, also all economic, social, societal and ethical consequences. This is a first success. But it isn’t necessarily a sustainable success.”

Third, at the time of writing (December 2021), the balance between inhibiting movement and its safeguarding also informed debates about the state of ‘critical infrastructures,’ such as energy and water provision or the police forces. As a result of the Omicron variant with its increased contagiousness, those were seen as endangered regarding their capability to be quickly and flexibly mobilized.^9^ Finally, the governing of the pandemic through infrastructures also crucially encompassed digital communication, such as the ‘Coronavirus Warning App’ launched by the federal government, which was devised to inform its users about potential virus contraction risks by means of tracing their encounters with infected persons.^10^ The infrastructural politics of pandemic governance thus relied heavily on the biopolitical rationality of securing institutions and processes involved in, and affected by, the spread and the containment of the virus in the population.

At the same time, the oscillation between a politics of health security through immobilization and contact reductions and a politics of securing the viability of pandemic-related infrastructures was modulated by a third element, to be described as a politics of invoking (presumably) safe spaces. This concerned in particular the invocation of the ‘home’ as a place where to remain in safety and security, as opposed to the public where the measures of social distancing could, as was implied, never be fully secured. ‘Staying at home’ became the often-reproduced call of the Social Democrats, the Christian Democrats, and also the Green Party, which in their public campaigns underscored this call with visuals representing peaceful domesticity.^11^ Moreover, the crucial significance of the ‘home’ resulted from it being charged with tasks that would normally presuppose some sort of publicness—especially going to work and going to school, but also going shopping or going to the doctor. On 19 January 2021, federal and state governments decided together to oblige employers to enable working from home wherever possible.^12^ Through digital means, the ‘home’ was thus upgraded from a private space to a space that allowed compensating for the suspension and absence of a variety of public spaces (of work, of education, of shopping, of entertainment, etc.). In other words, the invocation of the ‘home’ was meant to vouch not only for health security through guaranteeing (as was hoped) social distancing, but also for the security of life-as-normal which could (as was claimed) be largely replicated in digital settings.

The policies of pandemic security in Germany thus focused on an infrastructural logic, in particular, the balancing between the interruption of circulation and its maintenance through other means, with the ‘home’ as a crucial hub, namely, a private, hence, ‘safe’ space which still could assume crucial social functions pertaining to the very continuation of society. The German government’s perceptions of a multilayered security crisis through the lens of infrastructures (the control of circulation of human and viral populations) thus operated through a twofold invocation of the population as the carrier of the pandemic—an invocation that can be paralleled with a combination of Foucault’s modalities of governmental and disciplinary power. Mobilities and their interruptions through travel bans, closed borders, lockdowns, vaccination status conditionalities on participation in public life, and the lifting of some or all these measures after a wave was survived, were devised to calibrate the relationship between human and viral populations—a point clearly preordained through Foucault’s characterizations of the rise of ‘security’ as a concept to control populations through the use of their very own statistical and stochastic features (Foucault 2009, pp. 1–86).

At the same time, the invocation of the ‘home’ as an (allegedly) safe place, which still allowed to take on additional functions such as schooling and working, clearly followed another dimension of infrastructural power. This dimension was not so much explicated in Foucault’s studies on governmentality and biopolitics as in *Discipline and Punish* (Foucault 1995), where he argues that the prison and the social milieu of delinquency it co-creates serve as a crucial component in the control of social mobilities (see Aradau 2016). Of course, this is not to condemn the politics of lockdown and quarantine as part of the “carceral archipelago” (Foucault 1995, p. 297), but to make a case for a more complex and encompassing notion of security infrastructures that, beyond the nexus of circulation and interruption (see Opitz 2016), also includes the aspects of stationarity and assemblage. Taken together, the largely governmentality-driven approach to anti-pandemic measures, to which some disciplinary aspects were added, was supposed to safeguard the viability of a liberal form of life, that is to say, a form of life crystallizing around a social milieu, epitomized in the ‘home,’ that stands for a certain lifestyle and at the same time is able and prepared to adopt social functions normally taken over by other institutions in liberal capitalism, like gainful employment, formal education, or consumption (Langenohl and Westermeier 2022).

To summarize, the German government, like European governments by and large, invested in an infrastructural politics of pandemic security, which introduced a combination of governmental (in Foucault’s sense) politics with some aspects of more disciplinary forms of power. Political discourse triumphed at any moment those measures led to a decrease in infection figures, affirming itself and the form of life that these politics were supposed to help secure. Yet at the same time, its effects were also represented in spectacular, and soon familiar, images of empty pedestrian areas, deserted airports and train stations, stadiums, classrooms and lecture halls, and other representations of ‘public life’ brought to an almost complete freeze.

The infrastructural politics of pandemic security thus were largely instrumental in making liberal life survive, not least in the form of a morally and functionally inflated notion of ‘home’ supposed to take over many of the public functions characterizing life in liberal democracies, and enabled to perform these tasks through digital connectivity. Yet at the same time, that politics produced vistas of public emptiness which it did not fill with its own presence. At this particular juncture, the infrastructural politics of security failed to make a connection between the securing of social connectivity and the invocation of any meaningful political collectivity (see Opitz and Tellmann 2015), despite the often-repeated invocations of a moral ‘we’ that, due to their solidarity and reasonableness, would overcome the pandemic. It is the argument of this paper that it was a political miscalculation of the German government, and other governments, to neglect the quality of these deserted spaces as spaces of political articulation, just waiting to be taken.

## Corona Counter-Publics: (Re-)Claiming Sovereign Visibility

Relationships between Foucault’s different modalities of power—governmentality, discipline, and sovereignty—are complex and far from unequivocal. While the chronological ordering both of his works and of the sequence of their publishing seem to indicate that Foucault wanted to accentuate the differences between sovereignty, discipline, and biopolitics (or governmentality) as modalities of power with clear historical points of gravitation, there is also explicit indication that these different modalities are not to be conceived of as mutually exclusive, but complementary and overlapping (Collier 2009). For the analysis presented here, however, it is important to point out that there is a categorical difference in the ways that Foucault conceived of the *visibility* and *publicity* of the three modalities of power. Discipline, epitomized in “incarceration” (Foucault 1995, p. 115), is outspokenly non-public and deliberately removed from observability. At most, it is visible in certain marginalized social milieus, controlled by the police and functioning together with the prison apparatus as a control of social mobility. In its turn, governmentality, rather than being a public phenomenon in itself, is constitutive of certain forms, including material forms, of publicness, as Foucault explains on the example of the built urban environment:

“What is a good street? A good street is one in which there is, of course, a circulation of what are called miasmas, and so diseases, and the street will have to be managed according to this necessary, although hardly desirable role. […] [T]he town will not be conceived or planned according to a static perception that would ensure the perfection of the function there and then, but will open onto a future that is not exactly controllable, not precisely measured and measurable, and a good town plan takes into account precisely what might happen.” (Foucault 2009:, pp. 19–20)

Later, Foucault theorized governmentality as a particular form of conduct of self and of others which is based on forms of subjectivization that have, first of all, a psychological and mental substrate (Foucault 2010).

In contrast to both discipline and governmentality, sovereign power is based on publicness. It works through the public spectacle in which it symbolically reaffirms itself, like in Foucault’s famous example of the public torturing to death of “Damiens the regicide” and the equally public dismemberment of his body (Foucault 1995, pp. 3–6). With only little exaggeration, one may say that the power modality that sovereignty represents has virtually no substrate apart from its public presence: The sovereign represents the people just as he represents order. When order is disrupted, it must be reaffirmed ceremonially.

It catches the eye how the infrastructural politics of pandemic security in Europe deliberately did *not* resort to the register of sovereign power. Apart from one or two iconic representations, like that of a military convoy in the streets of Bergamo that was related to the evacuation of the dead bodies of COVID-19 victims (and which was not authorized by the state but was a private shot), state institutions chose not to represent themselves in any particularly ‘sovereign’ way. The significant exception to this rule of state invisibility in what remained of the public were police forces turning against (sometimes, illegal, and often transgressive) public demonstrations of opponents to the measures.

It is exactly this latter example that shows how the infrastructural politics of pandemic governance went together with the rise of social constituencies that were not uniform in their social-structural position, motivation, or ideological convictions, but were capable of intensifying their protests over the course of the pandemic. In order to gauge the significance of this phenomenon, a conceptual analysis in terms of Foucault’s notion of sovereign power, and its relation to securitization theory, is helpful. Such an analysis will shed light not so much on the substantial motivations of the protesters, but rather on their structural position within a multi-dimensional array of modalities of security-related power that started shifting through the pandemic and the ways that governments in Europe responded to it.

To start with, the ‘classical’ Copenhagen school variety of securitization theory regards the “securitizing move” (Buzan et al. 1998, p. 25) as a fundamentally public act that announces a situation of existing or imminent vital threat to the society and the state. The illocutionary conceptualization of that speech act argues that securitization in itself changes the political status of its referent object (see the theoretical exposition in Vuori 2011). Although receiving criticism for not taking into account that relevant audiences have to be convinced of the saliency of the act (Balzacq 2005), the complex character of the securitizing move as an illocutionary speech act also has analytical merits, which are rather seldom remarked on. It carries the understanding that the securitizing move is based on political authority: not everyone can make such a move, but only figures who are politically authorized, in whatever way. This implies that a securitizing move cannot be ignored because it alters the array of political coordinates, irrespective of the question of whether it substantially convinces any audiences (Langenohl 2020). In turn, this has as a consequence that the securitizing move, as a communication that cannot be ignored, is capable of co-founding authority and sovereignty, of structuring public communication, and of aligning audiences. Put differently, it is principally able to *produce* audiences as it unfolds, maybe more than to *convince* them, which represents a deeper layer of the argument that the securitizing move is a “self-referential practice” (Buzan et al. 1998, p. 24; see also Huysmans 1998). To put the argument in a nutshell: the concept of the ‘securitizing move’ refers to a process of self-affirmation of political authority that works through the formatting of public political communication, in which authority flows precisely from the circular, and notably asymmetrical and hierarchical, difference between an authoritative speaker position and an audience position considered as passive.

At the same time, as Copenhagen school scholars have explained (Guzzini 2015), securitizing moves are not without risk. The gravest of these is a self-limitation regarding political options other than those that can be aligned with the “grammar of security” (Buzan et al. 1998, p. 33). As Guzzini (2015) has pointed out, this drawback of the securitizing move appeared so salient for Copenhagen scholars because they saw it epitomized in an international spiral of securitizations and counter-securitizations characteristic of the second Cold War of the 1970s and 1980s. In general, it has been remarked that the conceptualization of the securitizing move as invoking a state of emergency sits uneasily with the realities of the political public in liberal democracies, where securitizing moves might be openly and critically discussed even as they belong to the non-ignorable sort of political communication (Williams 2003).

Against the background of these remarks on the Copenhagen school concept of securitization and the securitizing move and their entanglement with the public, the specificities both of governments abstaining from securitizing moves as well as the considerably transgressive and threatening public protests against Corona-related measures become visible in their interdependency. On the one hand, governments in liberal democracies obviously hesitated to consistently use the ‘grammar of security’ even if they were of the opinion that the pandemic posed a threat to society (which has not been the case in all European countries at all times). This probably has to do with the two drawbacks of securitizing moves just mentioned. Securitizing moves tend to create positions from which political actors might find it difficult to row back once the grammar of security is invoked; and they might be challenged as to their adequacy (as has actually been done, even as the grammar of security did at no point dominate official political communication). Seen from this angle, the decision to abstain from securitizing moves and instead rely on the infrastructural politics of security—that is, the power of governmentality and biopolitics, combined with a dose of disciplining—appears to be a rational policy that promises to be able to flexibly adapt to the strong self-dynamics of the pandemic. At the same time, the refusal to activate the illocutionary grammar of security and to format public communication accordingly wasted an opportunity of political self-authorization in times of multiple crises.

On the other hand, the refusal of political authorities to invoke the grammar of security provided an opportunity for non-authorized actors and constituencies to appropriate it, and to attempt to use its illocutionary force for a gesture of political foundation and self-authorization. This could be observed in the development of public protests against Corona-related measures, politicians, and institutions involved in the fight against the pandemic, such as research institutes, vaccination centers, pharmacies, and general practitioners.^13^ Those protests have been described as increasingly radicalized, violence-prone, aggressive against the police, and directed against the fundamentals of public order and the state.^14^ Protest positionalities that initially insisted on freedom and constitutional rights were giving way to a more or less uniform rhetoric of threat. In these protests and on the social networks in which they flourished, the grammar of security dominated, even if in a sometimes vague and obtuse way. Slogans to be seen in anti-vaccination campaigns such as “Hands Off Our Children!”^15^ or “Stop the Vaccination Madness!”^16^ arose from social media communication where critics of vaccination mused about a coming civil war or ‘system change’ for which one ought to prepare, and opaque allusions to dark forces secretly steering the state were made.^17^ The rhetoric of danger, threat, and imminent radical transformations, directed against state institutions and any representatives of the ‘system,’ effectively painted a picture of state institutions as the enemy of (parts of) society. This representation could easily be combined with claims to sovereignty that denied the legitimacy of existing state institutions, as present in many protest constituencies such as the ‘Reichsbürger,’ preppers, or conspiracy ‘theorists’ whose mixture and subversion of many protest manifestations gave the Federal Office for the Protection of the Constitution (*Bundesamt für Verfassungsschutz*) a cause for concern.^18^ The spiral of (not only) rhetorical radicalization following from this was partly built into the logic of the grammar of security. It is difficult to retreat from it once it is invoked, all the more so as the protests followed no uniform strategy and had no single point of organizational gravity that might engage in the difficult work of desecuritization. However, even if there was such a central point, a full-fledged anti-state securitization would be completely in its political interest.

Thus, there was an interdependency between state institutions leaving the grammar of security to public political positions that repudiated the state and the radicalization of anti-pandemic measures which constituted direct threats not only to medically exposed individuals but for society and its democratic order as a whole. In order to explain the strength of these positions, one need not tackle the question of how strong the general population supported the protests on an attitudinal level. The protests unfolded their political force not within a stochastically structured social space, but within a symbolic space left without much regard by the infrastructural politics of pandemic security. Likewise, the vagueness and untenability of the protesters’ threat perceptions and security articulations did not make them any less powerful. Indeed, it was precisely this ‘empty’ character of the threat constructions (see Broecker 2021 on that point) that made possible the wild alliances of motivations and ideological positions that could be seen in the streets and on social media. In the example of the protests, the invocation of essential threats did indeed become visible in its illocutionary character. It performed an authority that it at the same time presupposed, intervening into the texture of public communication as something which cannot be ignored, and without the need and ambition to convince any ‘audience.’

## The Shortcomings of Governmentality: A Stocktaking

If the above-outlined interpretation of the selective modes of securitization employed by political institutions and their interdependency with the appropriation of the strategy of securitizing moves by the protest movement is correct, a number of evaluative statements regarding the effects of the political strategy to securitize the pandemic seem appropriate. All of them indicate that the exclusively infrastructural understanding of security in the pandemic, operating mainly through instruments of governmentality with a bit of disciplining, reached the limits of its political effectiveness.

First, through focusing exclusively on the infrastructural securitization of the pandemic, too much confidence was placed in the presence of liberal governmentalities and in matching subjectivities in the population. Any other subjectivities were merely politically rebutted as irresponsible and as threatening the success of the vaccination campaigns. The claim here is not that these other subjectivities were unproblematic from the point of view of the fight against the pandemic, but that they fell outside the control scope of governmental policies. In this sense, the narrowing of the politics of security to that of governmentality itself produced an ungovernable outside.

Second, and related to the first point, the infrastructural politics of security was based on an understanding of society as an entity that can be captured and represented through statistical and stochastic calculations and routines—the realm of the epidemiologist. To be sure, this understanding has proven to be impressively effective, given the general accurateness of epidemiological models and forecasts and the constant work on the calibration of these models as the pandemic took ever new turns. Yet, it fell short of the non-statistic and non-stochastic structuration of society and social communication, in particular, regarding public processes of political mobilization and radicalization. For instance, the invocation of the ‘home’ as a seriality of (allegedly, given strongly increased instances of domestic violence termed a ‘shadow pandemic’ by UN Women)^19^ safe places, which is reminiscent of a Thatcherite refutation of any notion of society and its disassociation into the total number of households modeled after nuclear families, turned a blind eye to the channels of communication and interchange that the liberal project of maintaining connectivity in the home presupposes in the first place. In this narrowed view, deviations from governmentality norms cannot be grasped in their socially structured quality, that is, as phenomena which are significant beyond their deviance from norms.

Third, political institutions and authorities failed to come up with any conception of public spaces, publicity, and political public communication that would be operable under conditions of lockdowns, social distancing, and shut physical public spaces. The politics of securing through governmentality and discipline instead implied a gradual suspension and reduction of public political activity. For instance, demonstrations were allowed only under conditions that ‘hygiene concepts’ were put in place by the organizers. They could, in principle, be disallowed and dissolved when those conditions were not met, or even when they were merely not expected to be met, as was the case with some demonstrations against the Corona-related measures (often organized by the so-called ‘Querdenker’ movement) in the summer of 2021.^20^ Again, this is neither to say that those conditions were irrational or opportunistic, nor that the hygienic misgivings about protests like those organized by the ‘Querdenker’ scene were unfounded. But it means that political actors and institutions were unable to see the pandemic as it was seen by the protesters, namely, as an *opportunity* for public political action and communication. There did not seem to exist the faintest idea as to how to mobilize a public majority against an aggressively public minority. The most recent epitomization of this epic failure of imagining a non-extremist political public in times of the pandemic was federal president Steinmeier’s praise of the “silent majority” of reasonable Germans, broadcast on Christmas (25 December 2021):

“I want to thank wholeheartedly the grand, often silent majority in our country that has been acting cautiously and responsibly for several months now. Because they have come to realize: We depend on one another more than ever—I depend on others and others depend on me.”^21^

Obviously, the head of state did not expect this silent majority to engage in anything more than a pandemic-conforming governmental self-conduct.

Fourth and finally, there was no conception in place regarding how to publicly represent political sovereignty under conditions of an aggressive pandemic. The sovereignty-wielding forces of the securitizing move were left to the radical and extremist system opposition. To be sure, medical experts, epidemiologists, and virologists were permitted (or urged, notably in medial contexts like talk shows) to play the role of the securitizer, but they directed their efforts understandably more toward the political institutions meandering from wave to wave than toward the extremist protesters. To avoid misunderstandings, the point to make here is not the authoritarian demand that the state engage in securitizing moves and the grammar of security with the aim to destroy its opponents. Instead, what is at stake is the diagnosis that, as a result of the exclusive focus on infrastructural invocations of security, state institutions have not bothered to address the problem of representing sovereignty in times of a most severe challenge. This constitutes a remarkable absence, given that national sovereignty, even according to the interpretation of the Copenhagen school, must still be regarded as the single most important incarnation of security as a value of its own (Wæver 1996). Instead, where governmentality obviously failed, more governmentality was preached—through appeals to solidarity, to regard for others, to informed self-interest, to reason. If sovereignty is a public claim based on contingency, as argued by Carl Schmitt (1934) and parts of contemporary political theory (Agamben 2002), then the protesters have adopted that argument much more effectively than the political institutions.

## Conclusion and Outlook

In the final analysis, the infrastructural politics of pandemic security performed by the German government incurred the risk that, under conditions of a global threat without modern precedence, matters of political sovereignty—that traditional Western core component of security—were left to the streets and a vitriolic digital ‘*foule*’ (Wiestler and Barth 2017).

It would be naïve, and normatively problematic, to recommend states and political institutions return to a classical politics of the securitizing move. Yet, some way of addressing the problem of security-as-sovereignty will have to be found, for two reasons. First, after Corona, other security issues that share with the pandemic its global and border transgressing character that per se puts sovereignty, if understood as national sovereignty, under stress—like climate change—will have to be tackled, prioritized, and securitized.^22^ That is to say, the notion of sovereignty will most likely far outlive its political functionality, in the form of a descriptor of crisis. Hence it should be taken into account by any near-future securitization strategy. Second, the concept of sovereignty, whatever its shortcomings and abuses, points to an agential quality beyond mere survival, much in line with the Welsh School’s conceptualization of security as more than the absence of threat—namely, as emancipation (Booth 1991). The German government did not do a bad job at securing liberal forms of life through the infrastructural politics of pandemic security—including, needless to say, the securing of blind spots of that form of life regarding social inequality and the marginalization of socially vulnerable groups. Yet, its failure consisted in not representing a liberal order as an achievement that needs to be secured and protected *together* with the biopolitical substrate of the population.

## Footnotes

1 I would like to thank two anonymous reviewers for their very insightful and detailed feedback on the paper.

2 https://www.mdr.de/nachrichten/deutschland/politik/impfgegner-fackelmarsch-koepping-kretschmer-bedrohung-100.html, last accessed 29 December 2021.

3 https://www.sueddeutsche.de/bayern/schweinfurt-corona-proteste-gewalt-1.5496346, last accessed 29 December 2021.

4 https://www.zdf.de/nachrichten/panorama/corona-tat-idar-oberstein-telegram-extremisten-100.html, last accessed 29 December 2021.

5 https://www.br.de/nachrichten/bayern/so-bereitet-sich-die-kritische-infrastruktur-auf-omikron-vor,Ss7w1n8, last accessed 29 December 2021.

6 https://www.bundesregierung.de/breg-de/aktuelles/fernsehansprache-von-bundeskanzlerin-angela-merkel-1732134, last accessed 20 June 2022 (author’s translation).

7 https://www.bundespraesident.de/SharedDocs/Reden/DE/Frank-Walter-Steinmeier/Reden/2020/04/200411-TV-Ansprache-Corona-Ostern.html, last accessed 20 June 2022 (author’s translation).

8 https://www.bundesregierung.de/breg-de/service/archiv/merkel-regierungserklaerung-1820302, last accessed 20 June 2022 (author’s translation).

9 https://taz.de/Omikron-Variante-gefaehrdet-Versorgung/!5821243/, last accessed 29 December 2021.

10 https://www.bundesregierung.de/breg-de/themen/corona-warn-app/corona-warn-app-englisch, last accessed 20 June 2022.

11 Cf. https://www.gruene-bw.de/gemeinsam-gegen-corona-wir-bleiben-zuhause/; https://www.cdusolingen.de/aktuelles/details/gemeinsam-gegen-corona-wir-bleiben-zuhause; https://www.spd-emlichheim.de/2020/03/21/gemeinsam-gegen-corona-2/, last accessed 17 June 2022.

12 https://www.bundesregierung.de/breg-de/aktuelles/bund-laender-beschluss-1841048, last accessed 20 June 2022.

13 https://www.dw.com/de/corona-impfgegner-bedrohen-%C3%A4rzte/a-59814527, last accessed 30 December 2021.

14 https://www.pnn.de/ueberregionales/wegen-aufrufen-zu-gewalt-und-protesten-justizministerium-will-telegram-als-soziales-netzwerk-regulieren/27886204.html, last accessedd 30 December 2021.

15 See, for instance, their petition call at https://www.openpetition.de/petition/blog/haende-weg-von-unseren-kindern, last accessed 29 December 2021.

16 Their petition can be viewed at https://www.change.org/p/regierung-stoppt-den-impfwahnsinn-c9412550-f5cb-4d72-82e4-821278302ba8, last accessed 29 December 2021.

17 Beuster, Mika (2021): Impfgegner streben “Parallelgesellschaft” an. Westerwälder Zeitung, 9. December 2021, p. 20.

18 https://www.zeit.de/news/2021-12/21/verfassungsschutz-corona-protestbewegung-zunehmend-rechts, last accessed 29 December 2021.

19 https://www.unwomen.org/en/news/in-focus/in-focus-gender-equality-in-covid-19-response/violence-against-women-during-covid-19, last accessed 20 June 2022.

20 https://www.rbb24.de/panorama/beitrag/2021/07/berlin-querdenker-demos-verboten-polizei-corona.html, last accessed 29 December 2021; see for a U.S. view on the ‘Querdenker’ movement https://www.washingtonpost.com/world/2020/12/09/germany-coronavirus-protests-radicalization-surve illance/, last accessed 17 June 2022.

21 https://www.bundespraesident.de/SharedDocs/Reden/DE/Frank-Walter-Steinmeier/Reden/2021/12/211225-Weihnachtsansprache-2021.html, last accessed 29 December 2021. Translation from German mine.

22 At the time of revising this paper (June 2022), most governments of the EU and/or NATO member states have rediscovered the notion of sovereignty, invoked as the inviolability of a state’s territorial integrity, in their reactions to Russia’s war against Ukraine.
